# Diagnosis of Human Visceral Pentastomiasis

**DOI:** 10.1371/journal.pntd.0000320

**Published:** 2009-02-24

**Authors:** Dennis Tappe, Dietrich W. Büttner

**Affiliations:** 1 Institute of Hygiene and Microbiology, University of Würzburg, Würzburg, Germany; 2 Department of Helminthology, Bernhard Nocht Institute for Tropical Medicine, Hamburg, Germany; George Washington University, United States of America

## Abstract

Visceral pentastomiasis in humans is caused by the larval stages (nymphs) of the arthropod-related tongue worms *Linguatula serrata*, *Armillifer armillatus*, *A. moniliformis*, *A. grandis*, and *Porocephalus crotali*. The majority of cases has been reported from Africa, Malaysia, and the Middle East, where visceral pentastomiasis may be an incidental finding in autopsies, and less often from China and Latin America. In Europe and North America, the disease is only rarely encountered in immigrants and long-term travelers, and the parasitic lesions may be confused with malignancies, leading to a delay in the correct diagnosis. Since clinical symptoms are variable and serological tests are not readily available, the diagnosis often relies on histopathological examinations. This laboratory symposium focuses on the diagnosis of this unusual parasitic disease and presents its risk factors and epidemiology.

## The Problem

**Figure 1:**
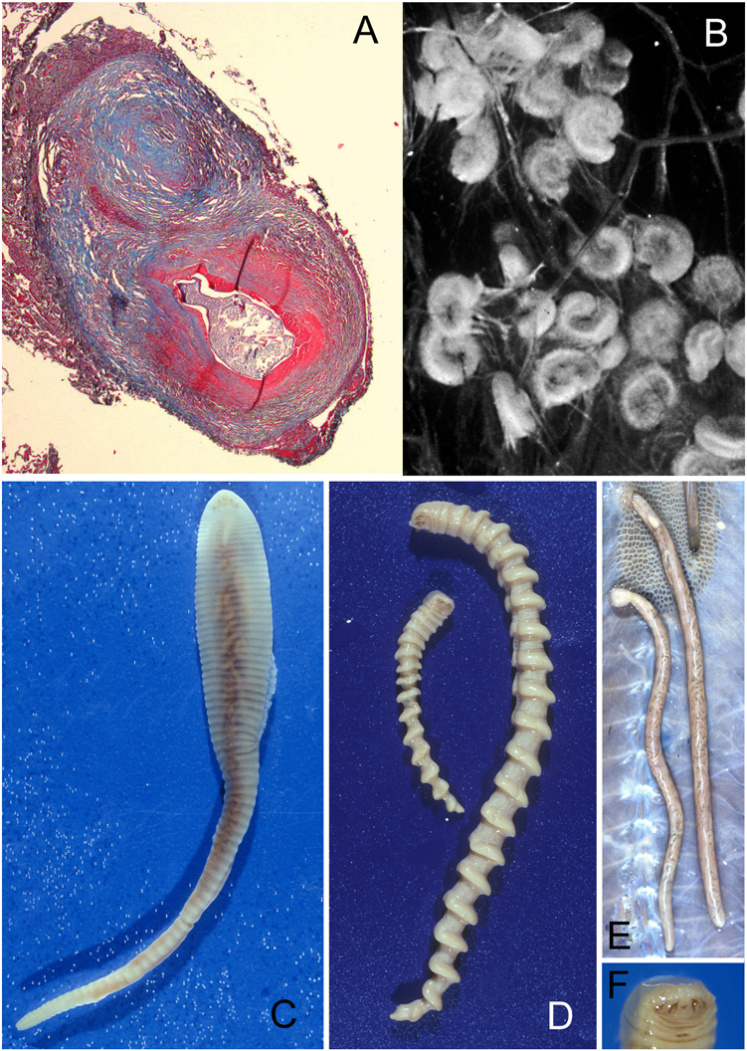
Nymphal and adult pentastomes. (A) Excised nodule on the pleural surface of the lung showing nymphal *L. serrata*. (B) Coiled nymphs of *Armillifer* sp. in simian omentum. (C) Adult female *L. serrata*. (D) Adult male (small) and female (large) of *Armillifer* sp. (E) Adult *P. crotali* in snake lung. (F) Anterior part of *Armillifer* with central mouth and four oral hooks.

In a recently published case [1], an immigrant from Kazakhstan was admitted to a hospital in Germany with persistent cough and night sweats. The patient had a normal white blood cell count (7,140 cells/µl) with mild eosinophilia (7%, 500 cells/µl). Serology for tissue-invasive helminths, e.g., *Ascaris*, *Toxocara*, *Strongyloides*, *Trichinella*, *Fasciola*, *Paragonimus*, *Schistosoma*, and *Echinococcus* spp. was negative, as were repeated stool and sputum examinations. A chest X ray revealed multiple lesions in both lungs, and pulmonary malignancy was suspected. The patient eventually underwent thoracotomy, and multiple nodules were excised that revealed exceptional parasitic structures (Figure 1A). Because of its peculiarity, the examination of the parasite responsible was lengthy, but finally a diagnosis of visceral (pulmonary) pentastomiasis due to the nymphs of *L. serrata* was made. Written consent of the patient was obtained for the publication of his clinical data and history.

## Tutorial

### What Is Visceral Pentastomiasis?

Visceral pentastomiasis is an unusual parasitic zoonosis caused by larval stages (nymphs) of several species of pentastomes (“tongue worms”), parasites that form a unique phylum with characteristics of both arthropods and annelids [2]. Species infecting humans belong to the families Linguatulidae, Armilliferidae, and Porocephalidae and have different geographic distributions (Table 1). More than 90% of human cases are caused by the nymphs of only two species, *L. serrata* and *A. armillatus* [3]. In humans, who accidentally serve as intermediate hosts, the infection develops when parasite ova are ingested from respiratory secretions or feces from the final hosts (dogs and other carnivores for *Linguatula*, several species of large snakes for *Armillifer* and *Porocephalus*). In the digestive tract of the human host, the minute four-legged primary larva hatches and invades the viscera. After encapsulation by host tissue and several molts, the infective larval stage develops (Figure 1B). In species infecting humans, the morphological appearance thereby changes and the nymphs finally resemble the adult legless vermiform pentastomes in shape (Figures 1C–1E).

**Table 1:** Characteristics of Pentastomes Infecting Humans.

Characteristics	*L. serrata*	*A. armillatus*	*A. grandis*	*A. moniliformis*	*P. crotali*
**Geographic distribution**	Cosmopolitan	West Africa	Central Africa	Southeast Asia	North, Central, and South America
**Natural definitive host (localization of adult parasite)**	Dogs, wolves (nasopharynx)	Pythons (respiratory tract)	Pythons (respiratory tract)	Cobras (respiratory tract)	Rattlesnakes (respiratory tract)
**Natural intermediate host**	Ruminants	Rodents, monkeys	Rodents, monkeys	Rodents, monkeys	Rodents, monkeys
**Characteristics of nymphs**	Flat, annulated; row of spines on each annulus; 4–6 mm body length	Cylindrical, spiral rings; 13–23 mm body length	Cylindrical, spiral rings; 9–15 mm body length	Cylindrical, spiral rings; 12–20 mm body length	Cylindrical, annulated; 12 mm body length

### What Are the Risk Factors for Infection, and How Can the Disease Be Prevented?

Close contact to dogs and their secretions predispose for infection with *L. serrata*, whereas people whose diet includes snake meat, workers at Asian snake-farms, snake keepers in zoos and pet shops, veterinarians, and owners of several species of pythons, vipers, cobras, and rattlesnakes may be exposed to ova of *Armillifer* and *Porocephalus*.

Visceral pentastomiasis can be prevented by proper hand washing after contact with dog saliva or feces and snake secretions or meat. Snake meat should be well cooked prior to consumption.

### Where May the Disease Be Contracted?

The highest prevalence of visceral pentastomiasis due to *L. serrata* has been reported from the Middle East [4], where high infection rates of dogs, the final host for *Linguatula*, have been noted. In Central and South America, sporadic cases have also been described [5]–[8]. The disease is rare in Europe, the United States, and China, where only a few cases have been reported [1], [9]–[13]. Infections with *Armillifer* spp. are most prevalent in West Africa, Central Africa [14]–[20], and Malaysia [21], where snakes, the final hosts of these parasites, are locally prepared for food. Autopsies performed in Nigeria revealed that in 33% of patients who died of malignancies, *Armillifer* was found [20]. A high frequency of 45% was also reported from a general autopsy study of aborigines in West Malaysia [21]. In Europe and North America the disease is rarely diagnosed, but it may be observed in immigrants from endemic areas and long-term travelers.

### What Are the Symptoms?

Symptoms depend on the organ system involved and result from the death of the nymphs or their migration. If only a few parasites are present, *L. serrata* may mimic hepatic or pulmonary malignancy clinically and on radiological assessments. Patients may develop abdominal pain, chronic cough, or night sweats [1],[8],[12]. In heavy infections with *Armillifer* spp., death may occur due to secondary septicemia, pneumonia, or severe enterocolitis [11],[19]. However, most human infections are asymptomatic, and the disease may be an incidental finding during routine medical consultation or at autopsy.

### Which Organ Systems Are Involved?

Pentastomid nymphs may be found throughout the peritoneal cavity, from which motile living parasites can be extracted during surgery or autopsy. Most parasites are located in the subperitoneal tissue around the liver, mesentery, spleen, and in the intestinal wall [22]. Infections of the liver parenchyma [5], [6], [8], [9]–[11] and abdominal lymph nodes [5],[11] are also frequently noted. Less often, parasites are found in the parenchyma of the lungs and on the pleural surface [1],[12]. Rarely, involvement of the heart [11], eye [7], and other organs has been described.

### How Can the Diagnosis Be Established?

**Figure 2:**
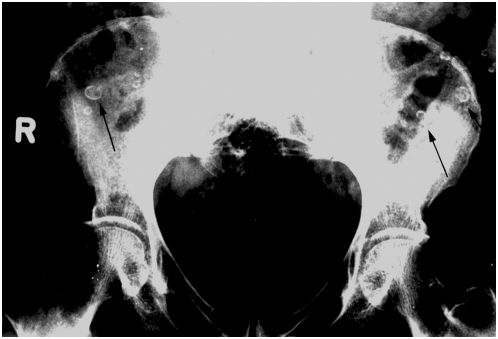
Radiograph of the pelvis from female patient with chronic lower abdominal pain. Calcified *Armillifer* nymphs (arrows) are shown in front of the iliac bones of a Nigerian patient. (Radiograph by courtesy of Klaus-J. Volkmer, Germany)

A radiological diagnosis is possible when calcified nymphs of *Armillifer* spp. and less often *L. serrata* are detected on pulmonal or abdominal radiographs, showing a horseshoe or C-shaped structure (Figure 2, [22]). There may be a mild eosinophilia. A few serological studies have been conducted [16]; however, no serological test is readily available, and no PCR test has been established for the diagnosis from biopsied tissues. Thus, visceral pentastomiasis often remains a histopathological diagnosis. The pentastome nymphs may be mistaken for other tissue-dwelling metazoan parasites, or, due to their peculiarity, the diagnosis may be delayed. The discrimination of pentastomes from other metazoa is needed to treat the patients who have infections with nematodes, cestodes, or trematodes.

### What Are the Morphologic Characteristics of Pentastome Nymphs?

**Figure 3:**
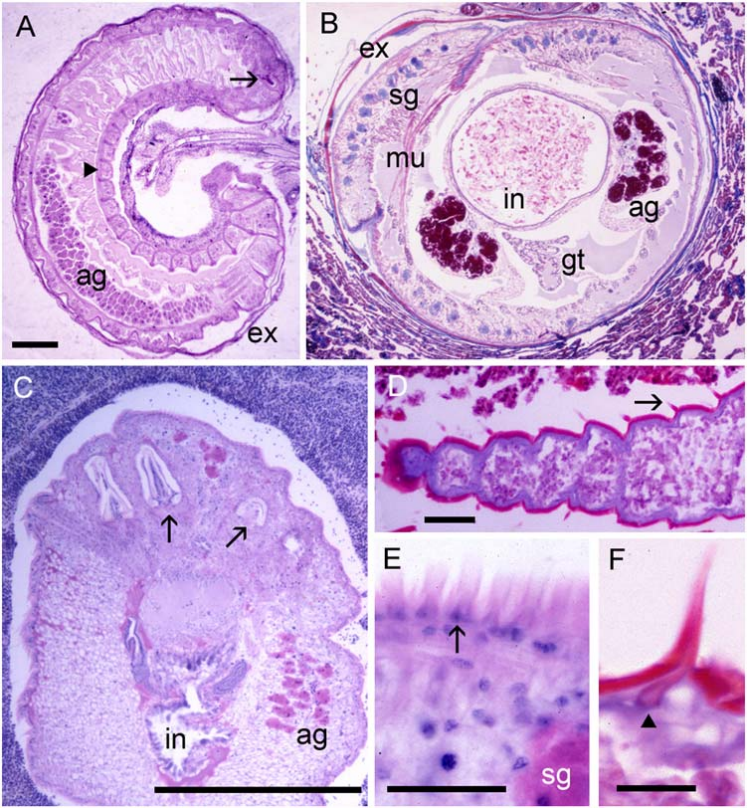
Sections through vital, well-preserved pentastomid nymphs. (A) Longitudinal section through coiled *Armillifer* sp. in the omentum with one oral hook visible (arrow). The cuticle shows sections of the prominent spirals (arrowhead). Note the shed cuticle (exuvia, ex) of the previous developmental stage in close contact to the parasite inside the capsule (ag, acidophilic glands). (B) Cross section of *Armillifer* sp. in the lung showing primordial genital tract (gt), red acidophilic (ag) and blue subcuticular glands (sg), intestine (in), muscles (mu), and exuvia (ex). There is not much host reaction. (C) Anterior portion of *L. serrata* nymph in a lymph node showing two pairs of oral hooks (arrows), intestine (in), and acidophilic glands (ag). (D) Part of a degenerated nymph showing cuticular annuli, which are responsible for the serrated aspect of this species. On each annulus, a spine is visible (arrow). (E) Close-up of row of spines (arrow). (F) Close-up of individual cuticular spine with separate inner structure (arrowhead). Scale bars: (A–C) = 500 µm; (D, E) = 40 µm; (F) = 20 µm. Hematoxylin and eosin (A, C); trichrome stain (all others).

**Figure 4:**
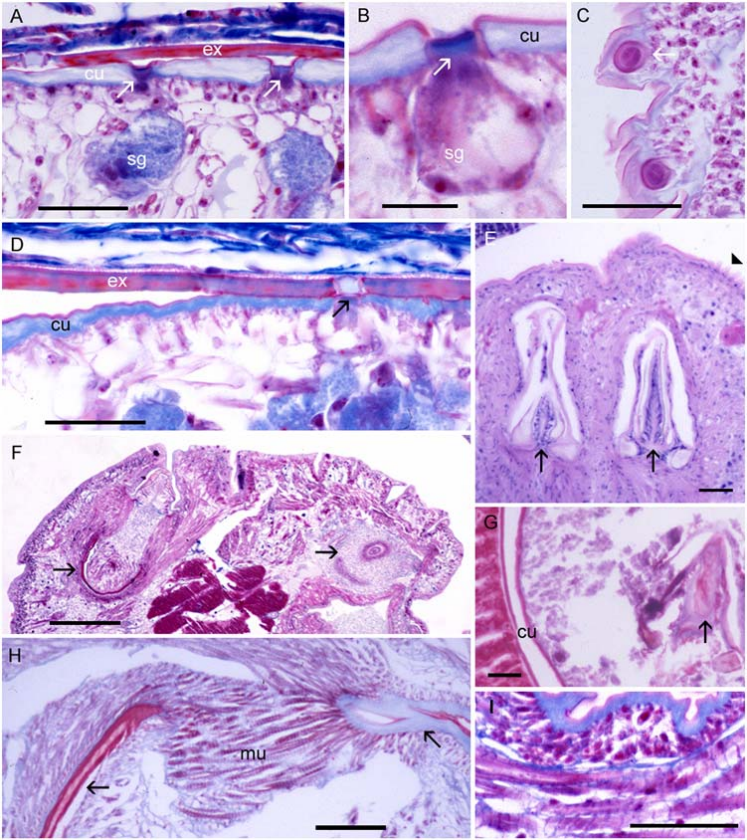
Body wall with cuticular openings and oral hooks of pentastome nymphs. (A–D) *Armillifer* nymphs with sclerotized openings (arrows) in the cuticle (cu) and the exuvia (ex). Subcuticular gland cells (sg) are also visible. (C) Cross section of two sclerotized openings. (E) Oral hooks (arrows) and spines (arrowhead) of *L. serrata*. (F) Anterior part of *A. armillatus* showing two oral hooks (arrows) and acidophilic glands (ag). (G) Degenerated *L. serrata* nymph with typical oral hook (arrow) and cuticle (cu) still visible. (H) Close-up of the base of an individual hook (arrows) of *A. armillatus* with fascicles of striated muscles (mu) inserting at the base. (I) Detail of subcuticular striated muscles. Scale bars: (B) = 10 µm; all others = 40 µm. Hematoxylin and eosin (E); trichrome stain (all others).

Mature infective nymphs of *Armillifer* spp. are cylindrical and have approximately 20 prominent spiral rings that contribute to a screw-like appearance as that of the adult parasites (Table 1). The nymphs of *A. grandis* are the smallest of the three *Armillifer* species. Those of *P. crotali* are cylindrical and annulated and have 38–40 body segments [23]. Nymphs of *L. serrata* are flat, slightly annulated, and have 72–92 body segments. All infective pentastomid nymphs possess a ventrally located mouth (Figure 1F), very typical large acidophilic glands in the anterior region, a digestive system, and primordial genital organs. The latter two may be seen on cross sections in well-preserved specimens (Figures 3B and 3C). Circumorally, two pairs of jointed chitinous hooks can be seen (Figures 1F and 3C). The combination of a centrally located mouth surrounded by four hooks evokes the impression of an organism with five mouths—hence the name “pentastomes.” Among the pentastomes observed in humans, only *L. serrata* has prominent spines attached to the parasite's cuticle. The spines measure from 32 µm in the anterior part of the nymph to 16 µm in the posterior parts. They are separated by a mean distance of 11 µm [7] and are placed on each body segment in a row. The chitinous cuticle of pentastome nymphs is penetrated by ring-like structures (Figures 4A–4D), which represent sclerotized openings of the ducts of subcuticular glands (Figures 4A, 4B, and 4D). Any routine stain will reveal the openings, but best results are achieved with Movat's pentachrome [24] or Masson's trichrome stain. Fascicles of striated muscle (Figure 4I) are found in the body wall, intermingled with the small subcuticular glands. In close contact to the parasite, the shed cuticle (exuvia) of the previous nymph can often be seen (Figures 3A, 3B, 4A, and 4D). A typical criterion for the diagnosis of pentastomes in tissue sections are the oral hooks (Figures 4E–4H). They can be retracted by fascicles of striated muscles that insert at the base (Figures 4F and 4H). In degradated nymphs, the hooks (Figure 4G) and remnants of the cuticle with sclerotized openings are often the only diagnostic features that remain. Occasionally the diagnosis can be based only on striated muscles (Figure 4I), provided that arthropods can be excluded.

### What Do Pentastome Lesions Look Like?

Macroscopically, the lesions are nodular or cyst-like. They are sometimes linked by a short stalk to the serosa or organ surface (Figure 3A, [11]). In infections with *L. serrata* they are called “*Linguatula* nodules” (Figure 1A) and have a size of 2–8 mm [11],[25]. Histologically, they represent granulomatous nodules, often with a target-like appearance due to the presence of fibrous or hyalinized concentric rings of connective tissue surrounding the parasite. Inside this capsule, legs and claws of the primary larva have disappeared, and the diagnostically significant, next-developmental-stage nymphs are coiled in a flat, C-shaped spiral. After several molts in the capsule, the infective nymphs resemble the adult pentastome in shape, but they are smaller. Detailed histopathologic descriptions of the surrounding tissue reactions in humans are scarce and usually only based on a few patients. Animal infection studies performed with various natural intermediate hosts have shown similar tissue reaction patterns. However, these patterns may not always be typical for accidental human intermediate hosts.

**Figure 5:**
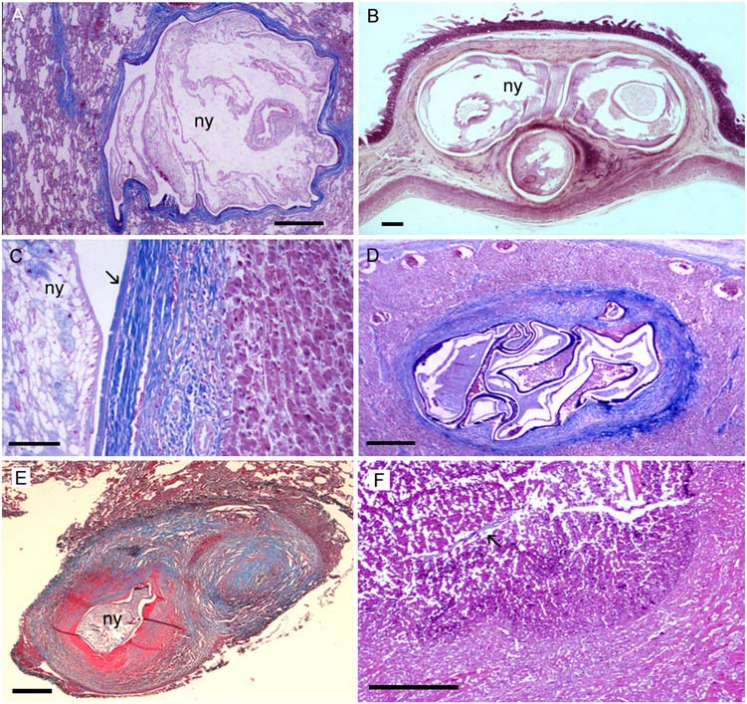
Different types of tissue reactions of human patients to vital, degenerated, and nearly absorbed pentastome nymphs. (A) Viable nymph (ny) of *A. armillatus* with thin fibrous capsule in the lung. (B) Three cross sections of one viable *A. armillatus* in the intestinal wall with very little cellular reaction. (C) Body wall of a well-preserved vital nymph (ny) of *A. armillatus* in the liver with exuvia (arrow), a thick fibrous capsule, and moderate infiltration of the adjacent liver tissue. (D) Dead and degenerated *A. armillatus* in a lymph node with a thick fibrous capsule. (E) *Linguatula* nodule with target-like appearance in human lung. In the center of the nodule, a dead degenerated nymph (ny) is visible. Due to the coiled shape of the nymph, two adjacent fibrous rings are discernable on this section. (F) Granulomatous scar with central amorphous mass in the lung. Remnants of the cuticle (arrow) of *L. serrata* are still left. Scale bars: (C) = 100 mm; all others = 500 µm. Hematoxylin and eosin (B); trichrome stain (all others).

In human patients, three types of pentastomid lesions have been described histologically [8],[11],[21]. In the first type, a viable nymph (Figures 5A–5C) is found in a cyst with little or no adjacent cellular infiltration of the host tissue, since the living nymph excretes only small amounts of antigenic compounds. Surrounding the parasite, usually a thin layer of homogenous, refractile, eosinophilic material of the exuvia is seen (Figures 5C and 5D, [24]). A narrow zone of epitheloid cells may adjoin the fibrous capsule (Figure 5C), peripheral to which are macrophages and, rarely, a few giant cells and lymphocytes. Usually only few or no eosinophils are seen [21]. Based on the morphology of the nymph, pentastomiasis can be diagnosed etiopathologically [11], and usually the pentastome genus or family can be identified in lesions of this type.

In the second and most common type of lesion in long-standing infections, the necrotic pentastomid granuloma [8],[11],[21], a dead nymph is found (Figures 5D and 5E). Large amounts of antigens are released from the dying nymph, and many immune cells are attracted. A central necrosis is surrounded by a thin wall of hyalinized epitheloid cells [24], followed by a large area of fibrous tissue with a few giant cells, macrophages, lymphocytes, plasma cells, and often many eosinophils. Concentric rings form the fibrous capsule with target-like appearance (Figure 1A). The fibrous rings may be calcified [21]. The parasite has disintegrated and is often calcified, but decay-refractory structures of the parasite, such as oral hooks or the chitinous cuticle, are still present. These remains may allow the diagnosis of pentastomiasis, but the genus of the pentastome responsible is often not determinable. Focal hemorrhages and congestion of blood vessels may be observed [1],[8]. In the liver, portal tract infiltration of eosinophils and lymphocytes may be seen [25], and degeneration of parenchymal cells surrounding the lesion may be evident.

In the third type, the granulomatous scar [11] or cuticle granuloma [21], typical structures of pentastomes are no longer found and no or little antigens are released. Only acellular, partly hyalinized fibrous tissue surrounding a central mass of amorphous or calcified material is visible (Figure 5F, [25]). In the concavity of the C-shaped fibrotic lesion lymphocytes have been described, but no eosinophils [25]. The periphery of the lesion is sharply demarcated from the surrounding parenchyma. The nymph has completely disintegrated and only small remnants of the parasite's cuticle may be left in the center (Figure 5F). It has been stated that the typical anatomical location of the lesion and its histological target-like appearance may be the basis for a presumptive diagnosis of pentastomiasis [11].

### How Should Infected Patients Be Treated?

In asymptomatic patients no treatment is necessary, since the parasites degenerate after approximately two years [7]. Only in symptomatic infections with numerous parasites may a surgical approach have to be considered. There is no antiparasitic chemotherapy available for pentastomiasis.

**Key Learning Points**

- Several species of pentastomes may infect human viscera, most often *L. serrata* and *A. armillatus*. The liver, peritoneum, lungs, pleura, and eyes are the organs most involved, and the infection may mimic malignancy clinically.
- Most infections are asymptomatic and an incidental finding. However, fatal consequences have been reported in a few cases.
- Diagnosis is mainly based on a characteristic radiograph or the histopathological examination of biopsied lesions. A correct diagnosis is necessary in order to rule out malignancy and to treat patients with trematodal, cestodal, or nematodal infections accordingly.
- The description of the morphological characteristics of pentastome nymphs, the anatomical distribution of the lesion, and the surrounding tissue reaction, as well as the geographic distribution of the different pentastome species and the patient's regional provenance, are the key components to diagnose visceral pentastomiasis.
- There is no medical treatment available, and in most cases no treatment is necessary at all. Only in heavy symptomatic infections may a surgical approach have to be considered.

## Supporting Information

Alternative Language Abstract S1 Translation of the Abstract into German by Dennis Tappe
(0.03 MB DOC)
