# Supplementary material for: Diagnosis of Human Visceral Pentastomiasis
Source: PLoS Negl Trop Dis. 2009 Feb 24;3(2):e320. doi: 10.1371/journal.pntd.0000320 (PMC2643528; doi:10.1371/journal.pntd.0000320)
Supplement: Alternative Language Abstract S1 — Translation of the Abstract into German by Dennis Tappe (0.03 MB DOC) [file pntd.0000320.s001.doc]

**Supporting Information File**

**Translation of the abstract** **of “Diagnosis of Human Visceral Pentastomiasis” into German by author Dennis Tappe**

Die viszerale Pentastomiasis des Menschen wird von den Larvenstadien (Nymphen) der Arthropoden-verwandten Zungenwürmer *Linguatula serrata*, *Armillifer armillatus, A. moniliformis*, *A. grandis* und *Porocephalus crotali* verursacht. Die Mehrzahl der Fälle wird aus Afrika, Malaysia und dem Nahen Osten berichtet, wo diese Erkrankung ein Zufallsbefund bei Obduktionen darstellen kann. Weniger häufig tritt die viszerale Pentastomiasis in China und Lateinamerika auf. Die Infektion ist in Europa und Nordamerika selten bei Immigranten und Langzeitreisenden zu finden; sie kann mit malignen Erkrankungen verwechselt werden und eine korrekte Diagnose kann verzögert werden. Da die klinischen Symptome variabel sind und serologische Tests nicht etabliert sind, hängt die Diagnose häufig von der histopathologischen Untersuchung ab. Dieses Laborsymposium konzentriert sich auf die Diagnostik dieser ungewöhnlichen parasitären Erkrankung und stellt die Risikofaktoren sowie die Epidemiologie vor.
